# Seasonal Change of Sediment Microbial Communities and Methane Emission in Young and Old Mangrove Forests in Xuan Thuy National Park

**DOI:** 10.4014/jmb.2311.11050

**Published:** 2023-12-30

**Authors:** Cuong Tu Ho, Unno Tatsuya, Son Giang Nguyen, Thi-Hanh Nguyen, Son Truong Dinh, Son Tho Le, Thi-Minh-Hanh Pham

**Affiliations:** 1 Graduate University of Science and Technology, Vietnam Academy of Science and Technology, Hanoi 10072, Vietnam; 2 Department of Microbiology, Chungbuk National University, Cheongju, Republic of Korea; 3 Institute of Ecology and Biological Resources, Vietnam Academy of Science and Technology, Ha Noi 10072, Vietnam; 4 Institute of Chemistry, Vietnam Academy of Science and Technology, Ha Noi, 10072, Vietnam; 5 Vietnam National University of Agriculture, Ha Noi, Vietnam; 6 College of Forestry Biotechnology, Vietnam National University of Forestry, Ha Noi, Vietnam; 7 Institute of Mechanics, Vietnam Academy of Science and Technology, Ha Noi, Vietnam

**Keywords:** Methane rates, mangrove stand-ages, microbial communities, illumina sequencing, NGS

## Abstract

Microbial communities in mangrove forests have recently been intensively investigated to explain the ecosystem function of mangroves. In this study, the soil microbial communities under young (<11 years-old) and old (>17 years-old) mangroves have been studied during dry and wet seasons. In addition, biogeochemical properties of sediments and methane emission from the two different mangrove ages were measured. The results showed that young and old mangrove soil microbial communities were significantly different on both seasons. Seasons seem to affect microbial communities more than the mangrove age does. Proteobacteria and Chloroflexi were two top abundant phyla showing >15%. Physio-chemical properties of sediment samples showed no significant difference between mangrove ages, seasons, nor depth levels, except for TOC showing significant difference between the two seasons. The methane emission rates from the mangroves varied depending on seasons and ages of the mangrove. However, this did not show significant correlation with the microbial community shifts, suggesting that abundance of methanogens was not the driving factor for mangrove soil microbial communities.

## Introduction

Mangrove forests were the shelter for living organisms in tropical and subtropical coastal zones. The composition of mangrove ecosystems is influenced by tidal forces as well as high concentrations of dissolved nutrients and decomposing organic matter [1]. The mangrove forests were estimated to contribute to 10–15% global coast sediment carbon storage and provide nutrients and habitats for living organism including microorganisms, micro-/marco-fauna, and migratory birds [2, 3]. Microorganisms are key players in the mangrove ecosystem processes including the decomposition of organic matter (leaves and trees) and cycling sulfur, phosphate as well as nitrogen. These processes involve a complex bacterial community that produces and degrades nitrogen, phosphorus, and carbon substances, forming the basis of the system’s food chain [1]. Thereby, recent studies have focused on the distribution pattern and potential biogeochemical functions in mangrove ecosystems [3-6] in order to link microbial distribution and diversity pattern to environmental factors for better understanding ecosystem functions and biogeochemical processes.

In Vietnam, mangroves grow in very different patterns and structure, and among them, the typical mangroves for Northern mangroves were in Xuan Thuy National Park (XTNP) comprising a mixture of plantation and naturally regenerating forests [7]. Three main species including *Kandelia obovata*, *Sonneratia caseolaris*, and *Rhizophora stylosa* were present in XTNP. *K. obovata* contributing to carbon stocks and carbon burial rates in the sediment of XTNP, accounting for about 95% of the plantings [8]. The mangrove ecosystems of the XTNP with a valuable ecological resource provide important habitats for many species of fishes, invertebrates, and waterfowl, which is also an essential stopover for migratory birds between northern and southern Asia [9].

Previous studies in XTNP mostly focused on the environmental factors and carbon burials and decomposition rates [8, 10-13]. It was found that the accumulated carbon in soil and roots of the planted *K. obovata* were 146.78 ± 3.87 mg organic carbon (OC) ha^-1^ and 12.67 ± 0.14 mg OC ha^-1^, respectively [8]. At the same time, Ha *et al*. [10] found that a significant amount of CO_2_ were emitted to the atmosphere as well. At the sediment-air interface, CO_2_ fluxes measured in the wet and dry seasons in mangroves were 140.55 ± 109.66 and 50.50 ± 31.64 mmol CO_2_ m^-2^ d^-1^, respectively. In these studies, the organic matter burial in mangrove forests is estimated to be stored for long-term as atmospheric CO_2_. On the other hand, it has not yet been well investigated if microbes in the mangrove sediments metabolize these organic matters and return them to the atmosphere as CH_4_. Previous study calculated that methane can offset the CO_2_ removed via carbon burial, partially offset blue carbon burial rates in mangrove sediments on average by 20% when authors applied the 20-year global warming potential [14]. Thus, CH_4_ emissions as well as microbial communities from the mangroves should be characterized for the blue carbon assessments and explanation.

In this study, microbial communities from sediments in young (<11 years-old mangroves) and old (>17 years-old) mangroves were characterized by Illumina sequencing as well as measuring methane emission and physicochemical properties of sediments. Here, the microbial communities from different level of sediments and methane emission in young and old mangrove forests during wet and dry seasons to understand how environmental factors affect microbial communities and methane emission rate.

## Materials and Methods

### Sampling Sites

**Fig. 1:**
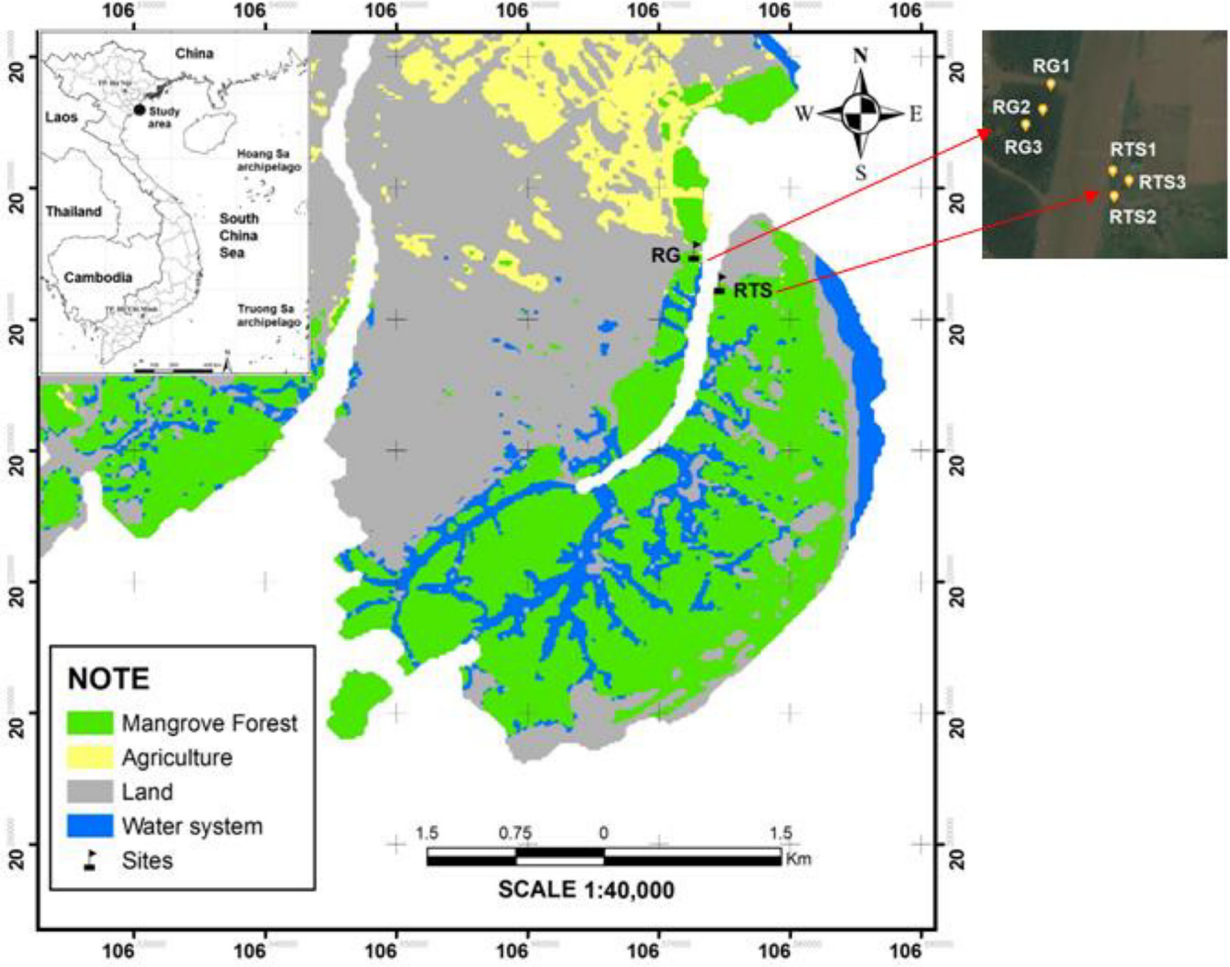
Map of sampling sites in Xuan Thuy National Park.

The investigation was carried out in part of an estuarine mangrove ecosystem of the XTNP in northern Vietnam, located at the northern part of the Ba Lat Estuary of the Red River which is the largest river in northern Vietnam (Fig. 1). The wetland area of the XTNP was 12,000 ha, of which about 3,000 ha are covered by mangrove forests that can be classified into natural and planted mangrove forests. The natural mangrove forests are mainly distributed in the northern area of the XTNP, dominated by the mangrove species, such as *Sonneratia caseolaris*, *Kandelia obovata*, *Aegiceras corniculatum*, and *Avicennia marina*. The planted mangrove forests are mainly distributed in the southern part of the XTNP, dominated by *K. obovata* [9]. Sediment samples were collected, and their properties were measured in a *K. obovata* forests distributed naturally along the Tra river. The mangroves were separated into two based on the tree heights (< 1.7 m of height for young mangrove is considered to be < 9 years-old, while>1.7 m of height is > 10 years-old) (Fig. S1, Table S1). The samples were collected at different depth (0-5 cm; 15-20 cm; and 35-40 cm). Three random sediment cores in the area of 30 × 30 cm^2^ were collected at three sites that was approximately 20 m far from each other) in the two forests. Field trips were carried out in dry season (April) and wet season (August) in 2019, and a total of 108 samples was collected.

### DNA Extraction, Amplification, Sequencing, and Data Processing

Sediment samples (1g of wet sediment) were dried in frozen state and stored at -20°C. DNA was extracted from approximately 250 mg dried sediments by using Power Soil DNA isolation kit (Qiagen GmbH, Germany). For microbial community analysis, V4 region of 16S rRNA gene was amplified using forward primer (AATGATACG GCGACCACCGAGATCTACAC-XXXXXXXX-TATGGTAATT-GT-GTGCCAGCMGCCGCGGTAA) and reverse primer (CAAGCAGAAGACGGCATACGAGAT-XXXXXXXX-AGTCAGTCAG-CC-GGACTACHVGGGTWT CTAAT) where X denotes nucleotides used to index each sample. Two microliters of the total DNA from each sample was used as a PCR template, and amplification was done in triplicates using iTaq mastermix 2X (INTRON Biotechnology Inc., Republic of Korea) with the following conditions: 95°C for 2 min; 33 cycles of 95°C for 20 s, 55°C for 15 s, and 72°C for 5 min; and 72°C for 10 min. Obtained PCR products were further purified using Qia quick purification kit (Qiagen GmbH). All purified DNAs were quantified, and equimolar amplicons were pooled and sent to Macrogen Inc. (Republic of Korea) for sequencing with Illumina’s MiSeq platform (2x300 bp). Raw sequence data were submitted to Genbank and deposited with project accession number PRJNA1007244.

Sequence analysis was conducted using Mothur software based on standard operation protocol (https://mothur.org/wiki/miseq_sop/) . Briefly, paired end reads were merged using make.contigs Mothur subroutine, then sequences containing low quality sequences (average=30) and ambiguous sequences (N) were removed using screen.seqs Mothur subroutine. Silva database (v128) was used to align the sequence, uchime Mothur subroutine was used to remove chimeric sequences, and RDP (v16) database was used for taxonomic classification with classify.seqs Mothur subroutine. For statistical analysis, all analyses were performed using R (R4.2.2) Vegan and Phyloseq packages following the pipeline (https://r-from-a-learners-perspective.readthedocs.io/en/latest/part5/). Alpha diversity was measured using Chao1, Shannon, and Inverse Simpson (E) indices. Non-metric multidimensional scaling (NMDS) based on Bray-Curtis distance was used to visualize differences in overall microbial community structures. The non-parametric test, permutational multivariate analysis of variance (Adonis), was performed based on Bray–Curtis distances to test dissimilarity of microbial communities among young and old mangroves. Analysis of variance (ANOVA) was performed to identify significant variation in alpha-diversity. Constrained analysis of principal coordinate (CAP) (or distance-based redundancy analysis) was performed to determine the relationships between microbial communities and environmental factors as previously suggested (Werner 2011).

### Methane Emission Rates and Measurement of Environmental Factors

Calculation of methane emission rates: The methane emission rates were measured for 3 sites in each mangrove forest. Methane concentrations were measured using a floating chamber system. The system included a handmade dark chamber (0.01292 m^3^; 0.06154 m^2^) of cylindrical polyvinyl chloride (PVC) connected to thermometer and fan (inside) (Fig. S1). The floating techniques were applied as described by [17]. Gas samples were collected three times (0, 10, and 30 min) at each sampling time and immediately injected into pre-evacuated 12 ml glass vial (15.5 mm diameter, Labco limited, UK). At each sampling site, gas samplings were performed in triplicate. All gas samples were collected in the high tide when the water level above the mangrove sediments in the range from 40 cm to 50 cm.

Methane concentrations in the samples were measured by a Gas Chromatography-Flame Ionization Detection (GC-FID). A flame ionization detector was set at 300°C, the oven temperature was controlled at 50°C, and helium (99.99%) was used as a carrier gas for methane concentration measurement. CH_4_ flux rate at the time of chamber closure was calculated through an equation previously described by Smith and Cresser [16].

Environmental factors analysis: 108 sediment samples were mixed based on the site sampling into 36 samples and were air-dried, ground, and sieved through a 2 mm sieve. The pH value and oxidation reduction potential (ORP/Eh) in soil samples were measured according to ASTM D4972 by using a HACH Sension 156E device. TN was quantified referring to the Devarda’alloys method. The ammonia converted from nitrate-nitrogen, nitrite-nitrogen, and organic nitrogen was liberated with strong alkaline, then quantified by colorimetry using Nessler’s reagent. To determine total phosphorus, sieved samples were digested with the mixtures of sulfuric acid, nitric acid, and perchloric acid. Concentrations of orthophosphate in the filtrates were measured by Vanadomolypdocolorimetric Acid Colorimetric method (APHA method 4500-P C). DOC was extracted from the soil samples using distilled water at a ratio of 1:10. DOC and TOC were ascertained via Walkley Black method (Soil quality - Determination of total organic carbon).

## Results and Discussion

### Variation in Methane Emission and Environmental Factors

**Fig. 2:**
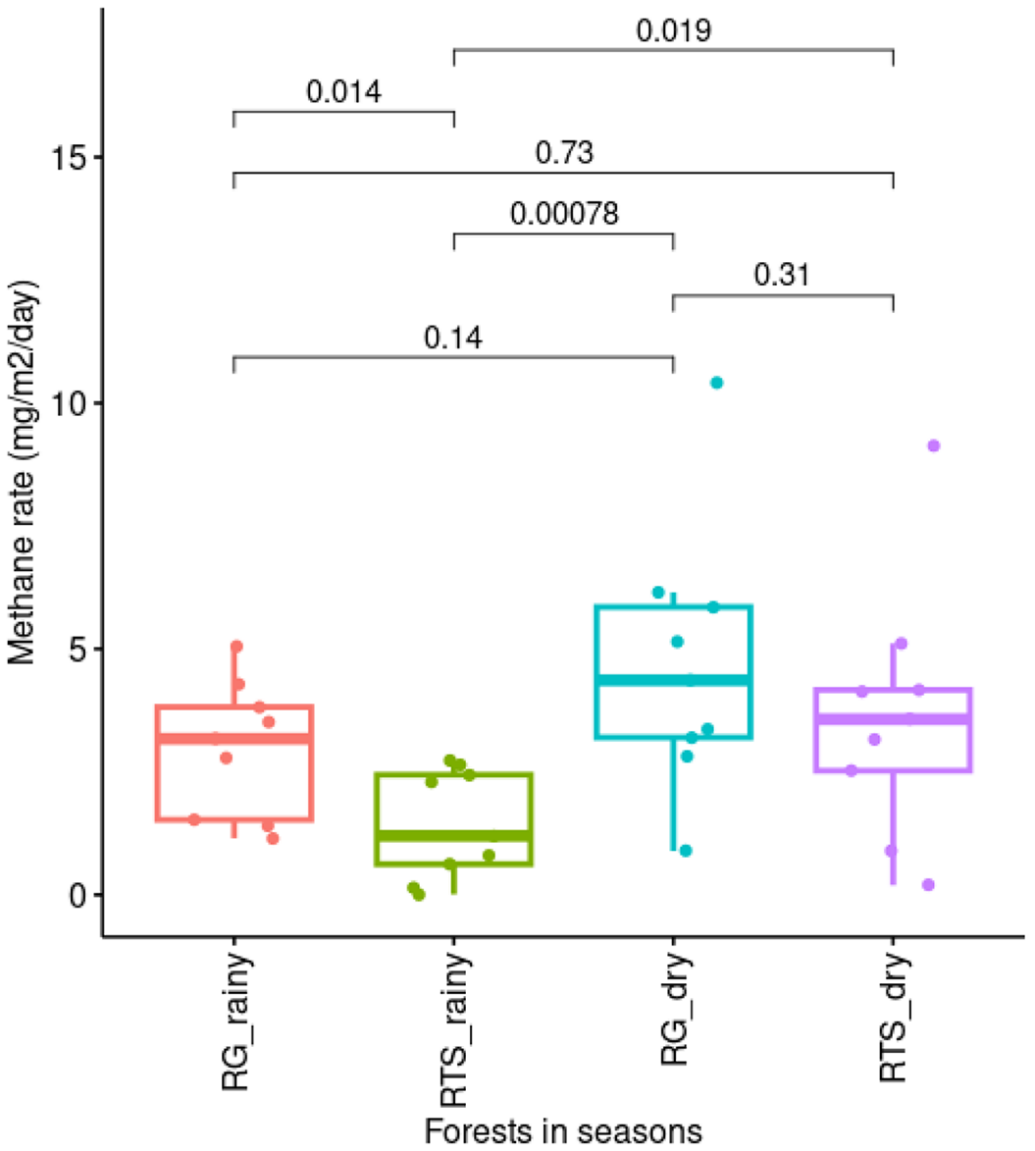
Methane emission rate in different mangrove forests in two (dry and wet) seasons in XTNP. (RG: old mangrove forest, RTS: young mangrove forest). The insert numbers are P-values between pairs based on ANOVA with posthoc Tukey HSD.

The level of methane emitted in young and old mangrove forests are different in wet and dry seasons. The mean value of methane emission rates in old mangrove varied from 2.97 to 4.69 mg m^-2^ day^-1^ for rainy and dry seasons, respectively, while it was 1.43 to 3.65 mg m^-2^ day^-1^ for rainy and dry seasons in young mangrove, respectively (Fig. 2). The statistical analysis revealed that the methane emission rates of young mangrove in rainy season were the lowest (*P*-values <0.05). The level of methane emission in old mangrove forest was not significantly different between the two seasons. Similar seasonal variations in greenhouse gas emission rates were observed in different estuarine wetlands in previous studies [10, 19, 20]. Allen *et al*. found that N_2_O and CH_4_ flux were the highest in April (Autumn) in the mangrove (*Avicennia marina*) forest [19]. Wang *et al*. also found that the CH_4_ flux exhibited seasonal variations in the mangrove forests with the highest flux (30.8 mg CH_4_ m^−2^ h^−1^) in summer [20]. Calculation of CO_2_ emission from sediment-air interface in planted mangrove showed that the fluxes in the wet season were significantly higher than in dried season, with mean values of 140.55 and 50.50 mmol CO_2_ m^-2^ day^-1^, respectively [10]. Studies also reported that the highest emission of CH_4_ typically occurred over the dried period as air temperatures rise. Another factor contributed to the increased CH_4_ emission can be the oxygen-consuming activities of aerobic microbes that rapidly cause a decline of redox potentials [20]. The soil moisture may be another important factor causing the seasonal variations in the methane fluxes. It should also be notified that naturally colonizing marsh had more methane emission than did the planted ones, likely due to the higher net primary productivity in the natural marsh. In our study, the methane emission from the old mangrove forest was higher than that of young mangrove forest, but not significantly.

In order to explain the methane emission from the mangroves, abiotic factors including pH, salinity, redox potential, TOC, total phosphorus, total nitrogen were measured at different depth of sediment in the mangroves. Our data (Fig. S2) show that these physicochemical parameters at different sediment depths did not show statistically significant differences, except for TOC, salinity, and pH in the sediments. In the rainy season, salinity seemed to be diluted at the shallow depth in old mangrove forests, while in young mangrove forest, high salinity was detected in the deep depth and the salinity was also diluted at the shallow and middle depth. In the dry season, the salinity was relatively stable and there was no significant difference between sediment depths. At the shallow depth of sediment in the old mangrove forest the pH was acidic, while it was neutral at the deeper depth. In the young mangrove forest, the pH was basic at the shallow depth, while the deep depth was more neutral. Our results also showed that TOC and DOC content at the deep depth were lower than two layers above in the old mangrove forest. The amount of TOC and DOC also significantly varied in different seasons.

**Fig. 3:**
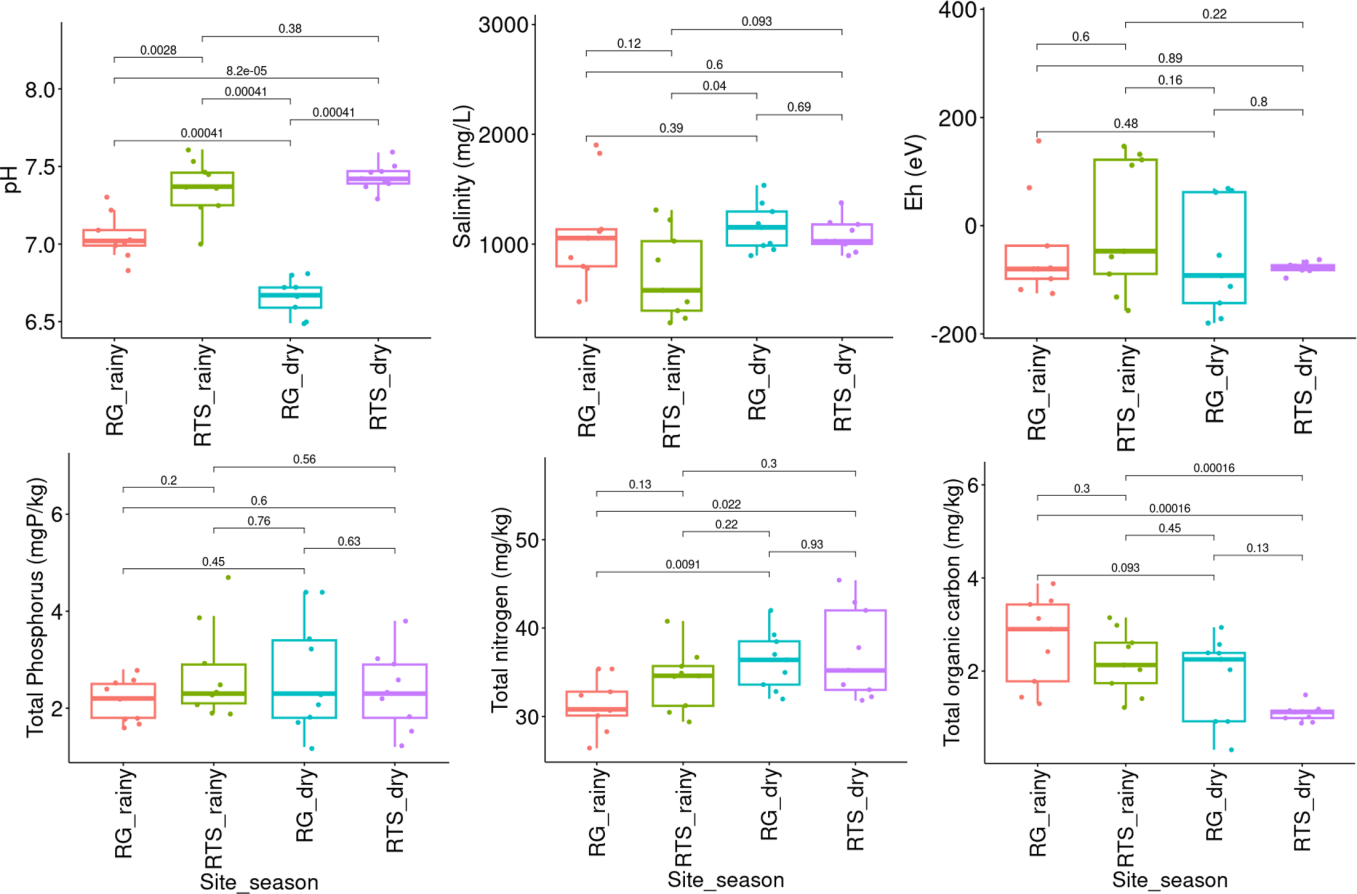
Environmental factors in different mangrove forests in two seasons (dry and rainy). The insert numbers are P-values between pairs based on ANOVA with post-hoc Tukey HSD.

The results presented in Fig. 3 statistically showed that the median values of total phosphorus and Eh change were not significantly different. Meanwhile, the mean value of total nitrogen content was only significantly different in the old mangrove sediment between the two seasons. The total nitrogen content in the dry season (36.3 mg kg^-1^) is significantly higher than in the rainy season (31.4 mg kg^-1^) in the old mangrove sediment. In young mangrove sediments, the total nitrogen content did not change significantly according to the season nor mangrove ages. It seems that rainy season influenced on sediment salinity of young mangrove sediments, showing the significantly lowest mean salinity (719.1 mg ^l-1^). The mean pH values of old mangrove sediments were significantly lower than the values of young mangrove sediments based on spatial and temporal factors. Finally, the concentration of TOC in the two mangrove sediments during rainy season was significantly higher than that in the dry season. Within the same seasons, this concentration was not statistically different for both young and old mangroves, it was probably due to the rainy season that brought more carbon to the terrestrial content. Our results were consistent with the measurement of pH and sediment salinity by Ha et. al. [10] in which the authors reported that pH and sediment salinity were decreased during the rainy season in mangrove forests. They also reported that the TOC in mangrove sediment were varied but not significant between the two seasons, which is likely because their sampling sites were in the south of XTNP where the mangroves sediments were hardly affected by Ba Lat estuary unlike our sampling sites. In our study, the amount of TOC in the rainy season was higher than that in dried season, implying our sampling sites received the terrestrial organic carbon from the Ba Lat estuary.

### Sediments Microbial Communities in Young and Old Mangrove in Two Seasons

In this study, we obtained a total of 6,253,779 reads. In this study, more than 100,000 reads were obtained from most of the samples. Our results showed almost equal microbial community structure within the triplicates (data not shown), thus these triplicates were pooled and regarded as one dataset, which reduced the number of microbial community data from 108 to 36. At the dissimilarity 0.03, we were able to assign operational taxonomic units (OTUs) to more than 80% of sequences (Table S2).

**Fig. 4:**
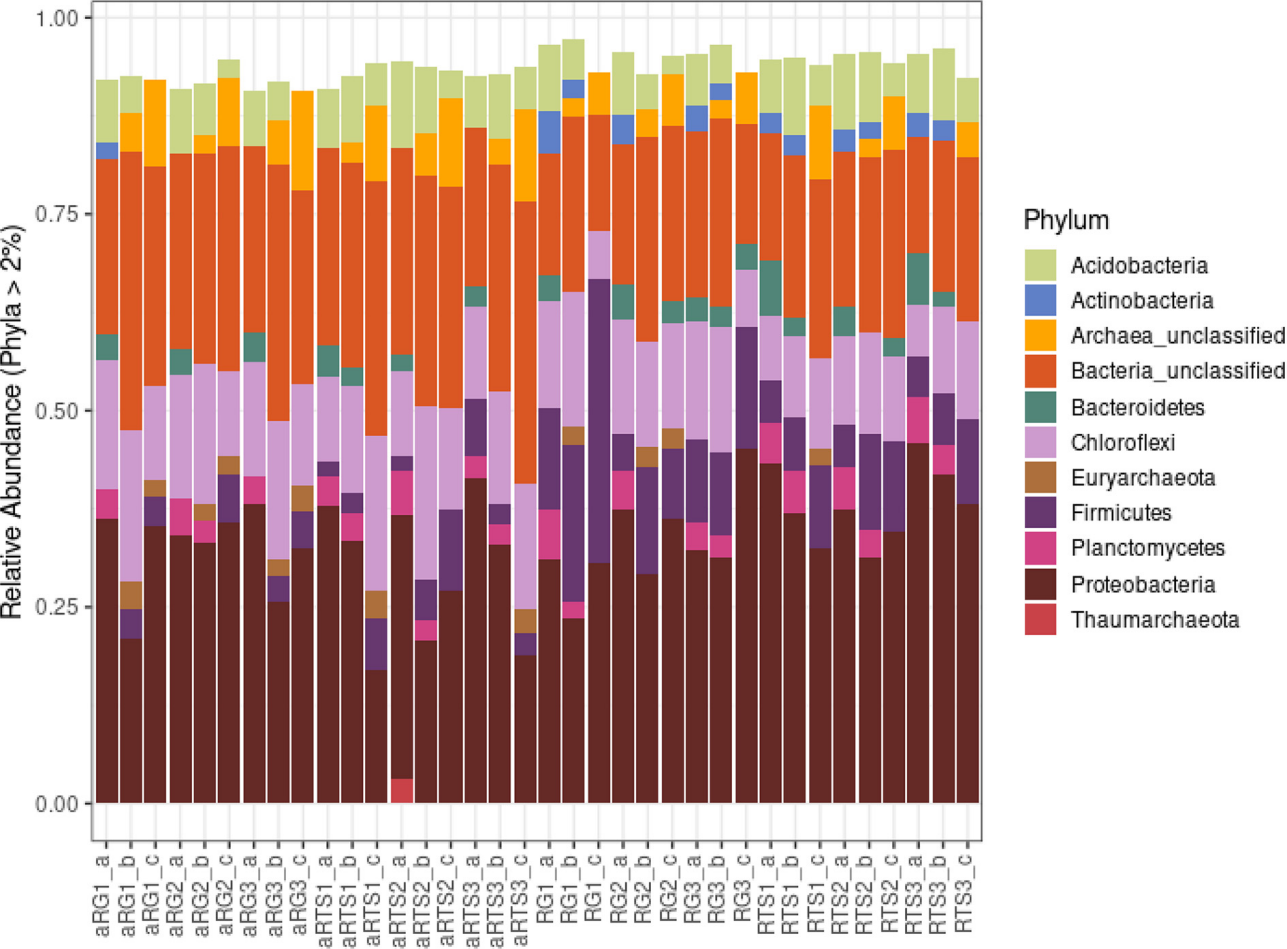
Dominant relative abundance (>2%) of microbial communities sediments in XTNP in two seasons (a_: rainy season).

**Fig. 5:**
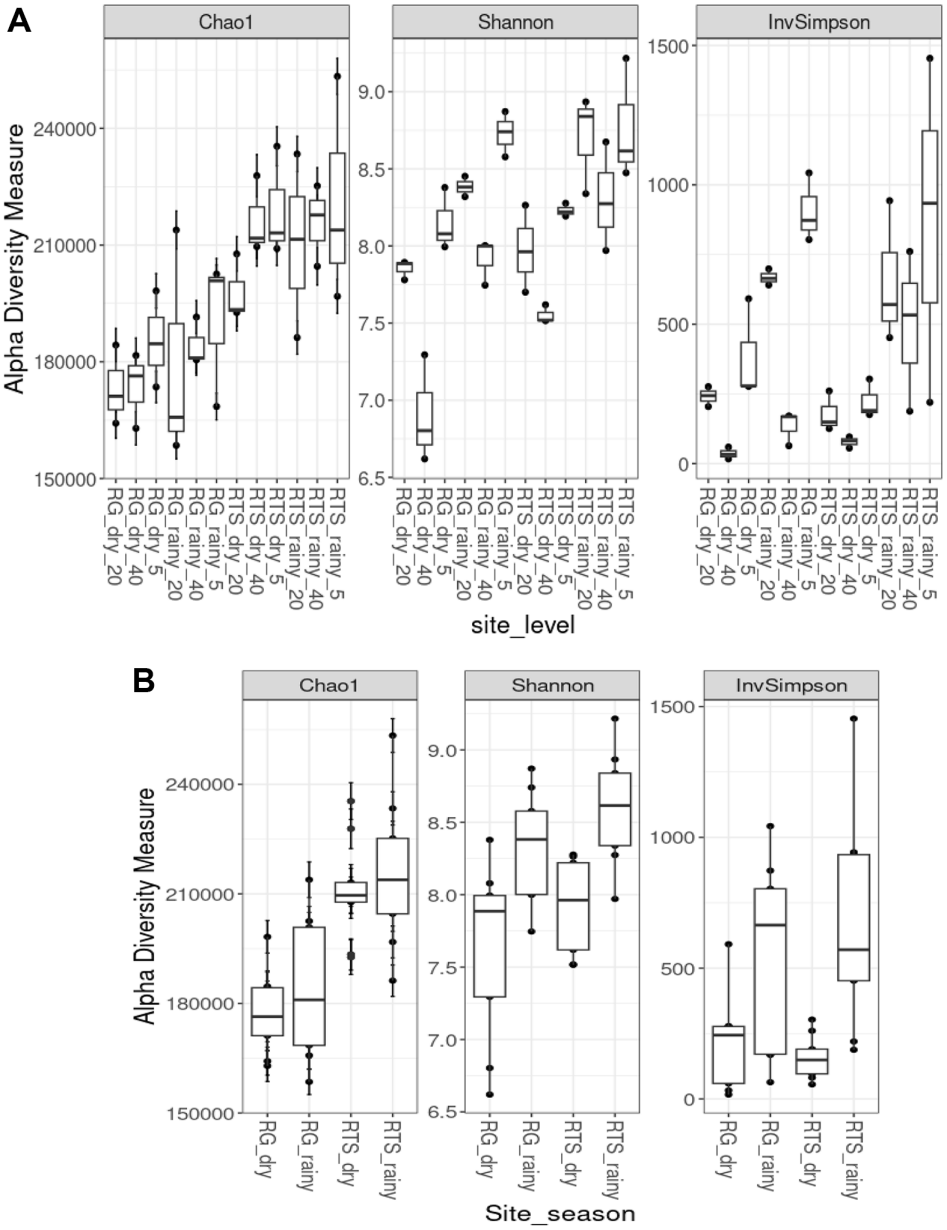
Diversity indices (Chao1, Shannon, Inverse Simpson) of microbial communities in XTNP; A) microbial communities at different depth of sites, B) microbial communities at different mangrove forests.

We observed a total of 45 phyla. We selected phyla with relative abundance higher than 2% and summarized in Fig. 4. The most abundant phyla in all communities include Proteobacteria and Chloroflexi, although a large number of bacteria belonged to the Bacteria_unclassified. On the other hand, dominant Acidobacteria (>2%) was observed in most of sediment samples while Actinomycetes were dominant in mangrove sediment during dry season. Overall, distribution of phyla in the sediments varied among samples even with the different depths within the same sampling sites. The alpha diversity analysis (Chao1, Shannon, and inverse Simpson) also confirmed the variation of the microbial communities at different depth of mangrove sediments, however, pairwise comparisons using Wilcoxon rank-sum test (Mann-Whitney) did not show significant differences for alpha diversity indices,(*P*-values = 1 for all pairs) (Fig. 5A). The alpha diversity indices were significantly different between the two seasons, particularly these indices were lower in dry seasons (Fig. 5B) while the mangrove age had no significant impact on these indices (except from Chao1).

**Fig. 6:**
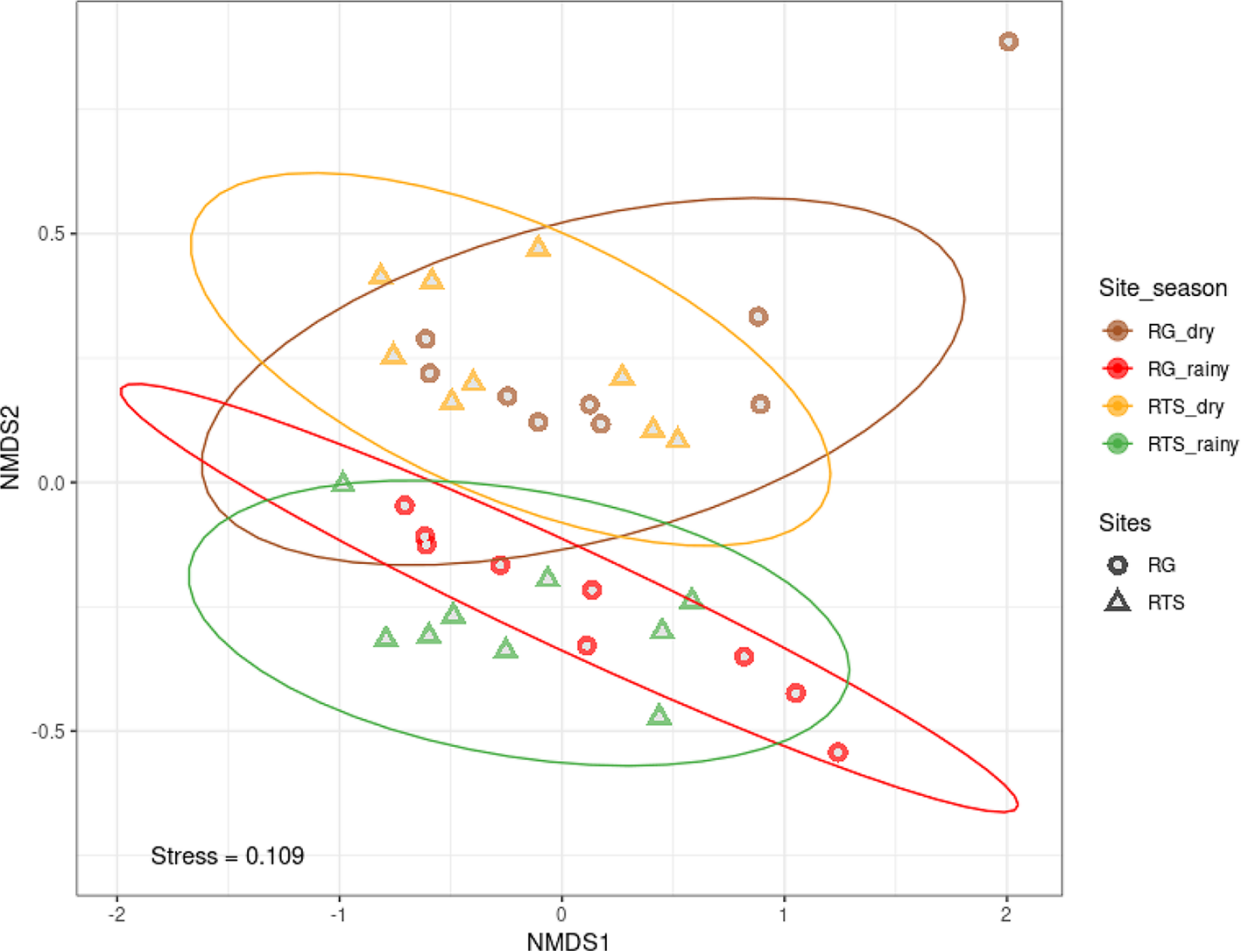
NMDS biplot based on Bray-Curtis distance demonstrating the different microbial communities based on sampling sites and seasons.

Fig. 6 describes the result of unconstrained analysis of these 36 microbial communities based on NMDS. The young or old mangrove microbial communities were completely separated from each other with different centroids, confirmed by testing the multivariate permutation on the distance matrix between the communities by Bray-curtis method. This difference was statistically significant with *P*-value <0.05 (Table 1). Table 1 also shows that the old mangrove microbial communities in dry season was significantly different from other communities. Meanwhile, the young mangrove microbial community in the rainy season was not significantly different from that of old mangrove in the rainy season. Overall, young and old mangrove microbial communities were completely different in the dry season, whereas the rainy season had shifted the community structures of these two mangrove sediments.

In previous studies, two classes Deltaproteobacteria and Gammaproteobacteria belonging to Protobacteria phylum were reported as the first dominant bacterial groups in rhizosphere sediments from many mangrove species [21-24]. The second most dominant phylum except from unknown bacteria in our study was Chloroflexi, that is consistent with the result found in three mangrove species sediments in Beilun Estuary [25]. Bacteroidetes are reported to be prevalent in tidal mudflats or near-shore sediments where *Rhizophora mangle*, *Avicennias chaueriana*, and *Laguncularia racemosa* are located in Guanabara Bay (Rio de Janeiro, Brazil) [26], which was observed in our study on both young and old mangrove sediments but not in all the depth. Consistent with previous reports, bacteria from the phyla Acidobacteria and Firmicutes were also found in the mangrove sediments in both seasons. Interestingly, Actinobacteria accounting for a large number in the microbial community structure in sediments in the Red Sea [23] were dominant in dry season but not in rainy season. In addition, Wu *et al*. reported that bacterial communities were not varied depending on the sediment depth, and the bacterial communities could be grouped based on the mangrove species [25]. In our study, the analysis of alpha diversity indices showed no significant difference in microbial community structure depending on the sampling depth.

### Microbial Community of Mangrove Sediments in Relation to Environmental Factors and Methane Emission

**Fig. 7:**
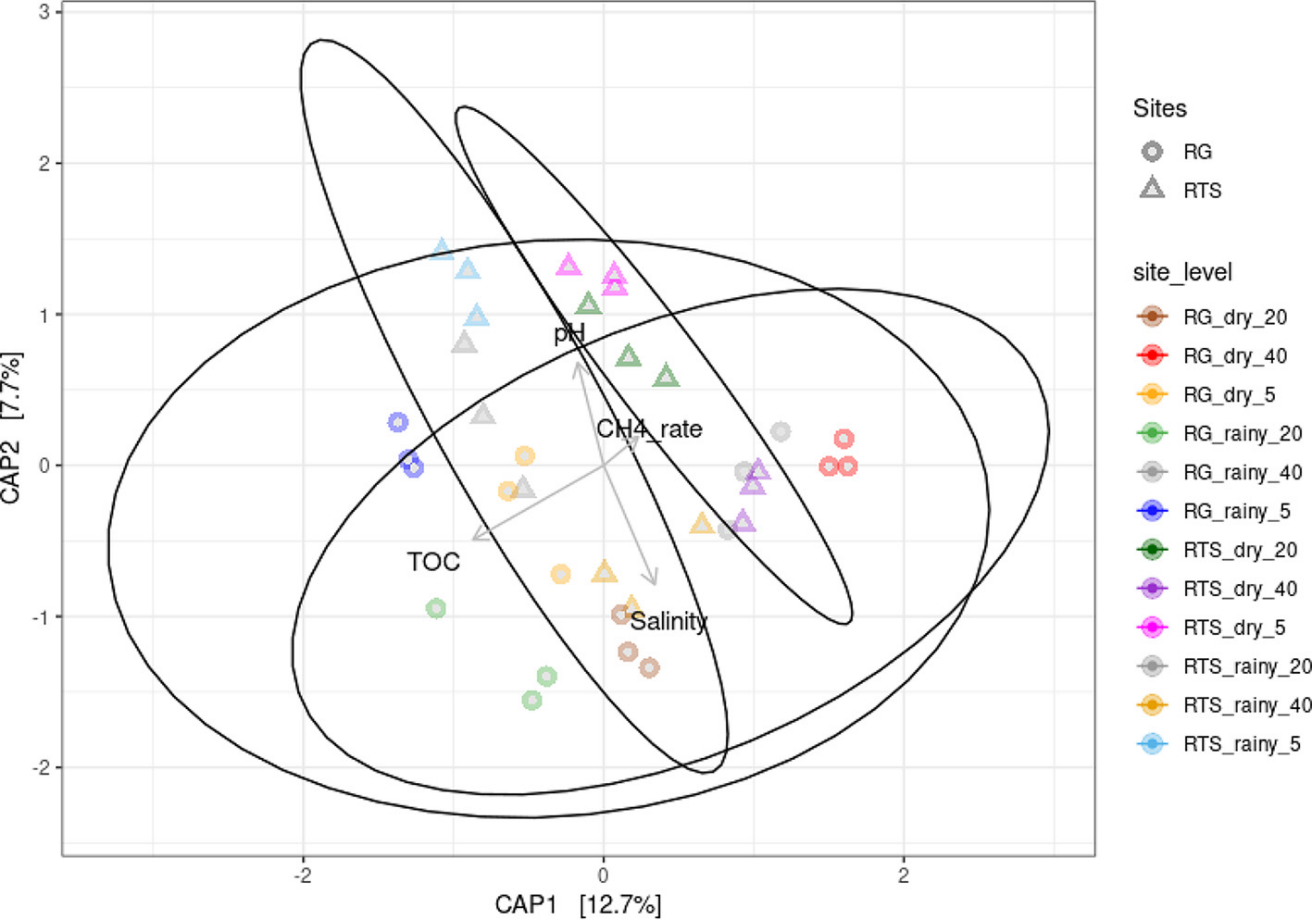
Constrained correspondence analysis (CCA) biplot of OTUs with mean relative abundance over 0.01% and environmental factors.

The relationship between environmental factors of sediments, methane emission rate, and microbial communities of mangrove sediments in two seasons was investigated by constrained analysis (CA). CA with linear model of distance matrix calculated by Bray-Curtis with four high loading factors including pH, salinity, total organic carbon (TOC), and methane emission rate (CH_4__rate) was applied. The results are presented in Fig. 7, in which, 20.4% of the data are plotted along the two constrained analysis of principal coordinate (CAP) axes. In addition, ANOVA using permutation showed that bacterial communities affected by environmental variables based on the given model were statistically significant (*p* = 0.001 with 999 permutation). The pH, salinity, TOC, and CH_4__rate were chosen for ordination because their loadings were the highest. The proportion of total inertia was small, approximately 27.29%, due to complex variation presented in a graph of two dimensions. When comparing with NMDS biplot, it was found that the constrained projection along environmental gradients Fig. 7 successfully grouped into four communities with different centroids as observed in Fig. 6.

Three environmental factors (pH, salinity, and TOC) were correlated with microbial communities of mangrove sediments. Fig. 7 also demonstrated that microbial communities of mangrove sediments were tightly correlated with TOC (R^2^ = 0.99, *p* = 0.001). The factor was positively correlated with rainy young and old mangroves, and dry old mangroves but negatively with dry young mangroves. The salinity significantly affected microbial community (R^2^ = 0.74, *p* = 0.001). While pH and CH_4__rate had lower correlation with microbial community of sediments, particularly, CH_4__rate had no significant correlation. The correlation of TOC and salinity with the microbial communities was hypothesized that the seasonal variation via the water flux from Ba Lat river mouth changed these two factors, implying the factors of shaping the microbial communities for whole area. The dry young mangrove forests were reported to produce the highest input of litter, leaves, nitrogen, and phosphorus to the environment [13, 27]. It implies that the young mangrove forests in dry season was able to produce more carbon because of high temperature as well as their productivity.

Previous studies also proposed several factors shaping mangrove rhizosphere microbial communities. Organic matters in the mangrove roots have been suggested to play an important role in shaping the mangrove rhizosphere microbial communities. However, Gomes *et al*. recommended this phenomenon appeared to be plant species-specific [21]. Exudates from mangrove root were hypothesized to not only increase microbial activities but create suboxic and anoxic microenvironments to facilitate nitrogen fixation [28]. Three non-parametric tests based on the Bray–Curtis distance matrix of mangrove sediment microbial communities showed that the different mangrove species possessed significantly different microbial communities [24]. In addition, root exudates from different mangrove selected specific group at both taxonomic levels and the functional levels [23, 24]. In our study, correlation between OTUs and TOC in both young and old mangrove sediments demonstrated that TOC were correlated with more OTUs in old mangrove sediments than that in young mangrove sediments. In the young mangrove sediments, there are only two OTUs belonging to *Rheinheimera* sp. and *Haliea* sp. that were correlated with TOC, implying other factors drove microbial communities rather than organic matters in young mangrove sediments (Table S3). The occurrence of water oligotroph *Rheinheimera* sp. [29-31] correlated with TOC in young mangrove sediments suggested that water flow brought adapted genus to the sediment. The correlation analysis between OTUs and methane emission rates also demonstrated the differences between the young and old mangrove sediments microbial communities, where there were a number of OTUs (*Archaea unclassified*, *Anaerolineaceae unclassified*, *Gp17 unclassified*, *Betaproteobacteria unclassified*,) correlated with methane emission rates in young mangrove forests, whereas there were no OTUs correlated with methane emission rate in old mangrove forest (Table S3). It was reported that *Anaerolineaceae* and *Methanosaeta* adapted with alkanes environment for methane emission [32]. In our study, these groups only correlated with methane emission rates, where the litterfalls were accumulated higher in young mangrove sediments compared to that in old mangrove sediments. We hypothesized that microbial guilds of old mangrove sediments adapted TOC in the sediment and became more abundant than the other groups, meanwhile the young mangrove sediments with lower organic carbon that came mainly from mangrove biomass resulted in the less abundant group for organic matters but functional microbial guild for methane emission.

## Conclusion

The microbial communities in sediment of the different mangrove forests were driven by the seasonal changes. The concentration of TOC in sediment in rainy season was higher than that in dry season at both young and old mangrove sediments, implying the mangroves sediments received organic matters higher amount from water raising due to terrestrial water from Ba Lat estuary. The TOC and salinity in sediment are a driving force for the microbial community structures in young and old mangrove sediments in both dry and wet seasons. The dynamic young mangrove seems not to bring organic carbon to sediment but water level raising in rainy seasons. The methane emission rates were not correlated with microbial communities of sediment in different mangrove forests but with unknown *Archaea* and *Anaerolineaceae*. Therefore, related methanogens were key group but not abundant contributors in the microbial communities

## Supplemental Materials

Supplementary data for this paper are available on-line only at http://jmb.or.kr.



## Figures and Tables

**Table 1:** Statistical value of multivariable analysis of permutation between distance matrices of communities based on Bray-Curtis calculation method.

	RG_rainy	RG_dry	RTS_rainy	RTS_dry
RG_rainy	1	-	-	-
RG_dry	0.12*	1	-	-
RTS_rainy	0.09	0.17*	1	-
RTS_dry	0.17*	0.11*	0.15*	1

*P-value < 0.05
