# Supplementary material for: Seasonal Change of Sediment Microbial Communities and Methane Emission in Young and Old Mangrove Forests in Xuan Thuy National Park
Source: J Microbiol Biotechnol. 2023 Dec 30;34(3):580–8. doi: 10.4014/jmb.2311.11050 (PMC11016791; doi:10.4014/jmb.2311.11050)
Supplement: Supplementary file 1 [file jmb-34-3-580-supple.pdf]

J. Microbiol. Biotechnol.
https://doi.org/10.4014/jmb.2311.11050

Supplementary Tables and Figures

Table S1. Coordinate of sampling sites in two different mangrove forests.

Sampling sites	Latitude (ºNorth)	Altitude (ºEast)
RTS1	20°14'36.9"N	106°34'28.0"E
RTS2	20°14'35.1"N	106°34'28.1"E
RTS3	20°14'36.2"N	106°34'29.2"E
RG1	20°14'43.1"N	106°34'23.3"E
RG2	20°14'41.3"N	106°34'22.7"E
RG3	20°14'40.2"N	106°34'21.4"E

Table S2. Information of raw sequences of pooled samples at level cutoff of 0.03.

Cutoff Level	Samples	No of reads	Coverage	No of OTU
0.03	RG1_a	202262	0.906324	29528
0.03	RG1_b	193067	0.919935	24675
0.03	RG1_c	106349	0.931791	12536
0.03	RG2_a	174156	0.899446	27909
0.03	RG2_b	179099	0.911429	25214
0.03	RG2_c	114636	0.912035	16624
0.03	RG3_a	188880	0.911637	27023
0.03	RG3_b	196115	0.909762	28362
0.03	RG3_c	117818	0.914716	16199
0.03	RTS1_a	184830	0.861851	37576
0.03	RTS1_b	188475	0.865441	37292
0.03	RTS1_c	174467	0.890701	27950
0.03	RTS2_a	190317	0.852451	39470
0.03	RTS2_b	193641	0.878202	33695
0.03	RTS2_c	170074	0.879053	29951
0.03	RTS3_a	146702	0.861208	30513
0.03	RTS3_b	172799	0.870844	32885
0.03	RTS3_c	143578	0.882029	25115
0.03	aRG1_a	171480	0.899195	28389
0.03	aRG1_b	159895	0.906076	24583
0.03	aRG1_c	161874	0.911098	23998
0.03	aRG2_a	179165	0.885056	33670
0.03	aRG2_b	190844	0.911252	28059
0.03	aRG2_c	161458	0.908199	24305
0.03	aRG3_a	164229	0.867551	33458
0.03	aRG3_b	179142	0.884449	31412
0.03	aRG3_c	105923	0.884029	19121
0.03	aRTS1_a	190511	0.849421	44249
0.03	aRTS1_b	197674	0.853865	43766
0.03	aRTS1_c	181387	0.877874	32240
0.03	aRTS2_a	178317	0.865234	35233
0.03	aRTS2_b	203689	0.874137	37211
0.03	aRTS2_c	177225	0.874098	31980
0.03	aRTS3_a	229849	0.855449	49087
0.03	aRTS3_b	208657	0.847726	46669
0.03	aRTS3_c	175195	0.851794	37924

Table S3. Correlation between OTUs and TOC and methane rate in different microbial communities, (-) no correlation; (*) *p*-value < 0.05.

OTUs	Rainy young stands		Dry young stands		Rainy young stands		Dry old stands	
	TOC	CH4_rate	TOC	CH4_rate	TOC	CH4_rate	TOC	CH4_rate
Vibrio sp_1	-	-	-	-	-0.846**	-	-0.733*	-
Anaerolineaceae unclassified_7	-	-	-	-	0.673*	-	0.818**	-
Anaerolineaceae unclassified_25	-	-	-	-	0.697*	-	0.791*	-
Vibrio sp_30	-	-	-	-	-0.734*	-	-0.760**	-
Photobacterium sp_6	-	-	-	-	-0.903***	-	-	-
Romboutsia sp_17	-	-	-	-	-	-	-	-
Clostridium_XI sp_4	-	-	-	-	-	-	0.765*	-
Microbulbifer sp_5	-	-	-	-	-	-	0.827**	-
Deltaproteobacteria unclassified_11	-	-	-	-	0.773*	-	0.828**	-
Archaea unclassified_12	-	-0.739*	-	-	-0.856*	-	-0.847**	-
Desulfobacteraceae unclassified_13	-	-	-	-	-	-	0.722*	-
Chloroflexi unclassified_19	-	-	-	-	-	-	0.716*	-
Anaerolineaceae unclassified_20	-	0.670*	-	-	0.886**	-	0.925***	-
Thioprofundum sp_22	-	-	-	-	0.898**	-	0.895**	-
Anaerolineaceae unclassified_23	-	-	-	-	0.935***	-	0.913***	-
Gp21 unclassified_24	-	-	-	-	0.978***	-	0.856**	-

Gp17 unclassified_28	-	0.757*	-	-	0.951***	-	0.835**	-
Gammaproteobacteria unclassified_29	-	-	-	-	0.747*	-	0.710*	-
Betaproteobacteria unclassified_32	-	0.686*	-	-	0.942***	-	0.694*	-
Anaerolineaceae unclassified_33	-	-	-	0.672*	0.900***	-	0.814**	-
Gammaproteobacteria unclassified_36	-	-	-	-	0.940***	-	0.733*	-
Deltaproteobacteria unclassified_37	-	-	-	-	0.957***	-	0.866**	-
Anaerolineaceae unclassified_48	-	-	-	-	0.906***	-	0.818**	-
Anaerolineaceae unclassified_3	-	-	-	-	0.768*	-	-	-
Bacillus sp_10	-	-	-	-	-0.669*	-	-	-
Rheinheimera sp_27	-	-	0.770*	-	-0.722*	-	-	-
Haliea sp_31	-	-	0.763*	-	0.702*	-	-	-
Shewanella sp_39	-	-	-	-	-0.805**	-	-	-
Anaerolineaceae unclassified_46	-	-	-	-	0.794*	-	-	-
Marinobacterium sp_49	-	-	-	-	0.706*	-	-	-
Chloroflexi unclassified_15	-	-	-	-	-	-	-	-
Bacillales unclassified_35	-	-	-	-	-	-	-	-
Archaea unclassified_38	-	-	-	-0.713*	-	-	-	-

Fig. S1. Sampling activities at two different mangrove forests. (A) Methane gas sampling in young mangroves at high tide (**B)** Sampling sediment cores in old mangrove at low tide.

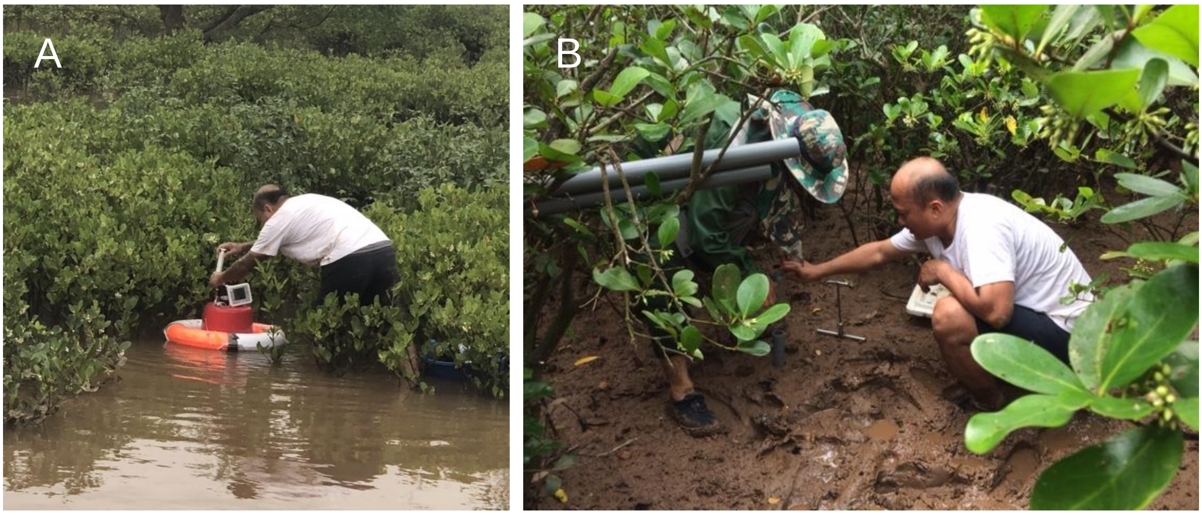

Fig. S2. Environmental factors at different sediment layers in different mangrove standage (RG: old mangrove forest; RTS: young mangrove forest) in two seasons (dry and rainy); ns: not significant, (.) significant with *P* values <0.05.

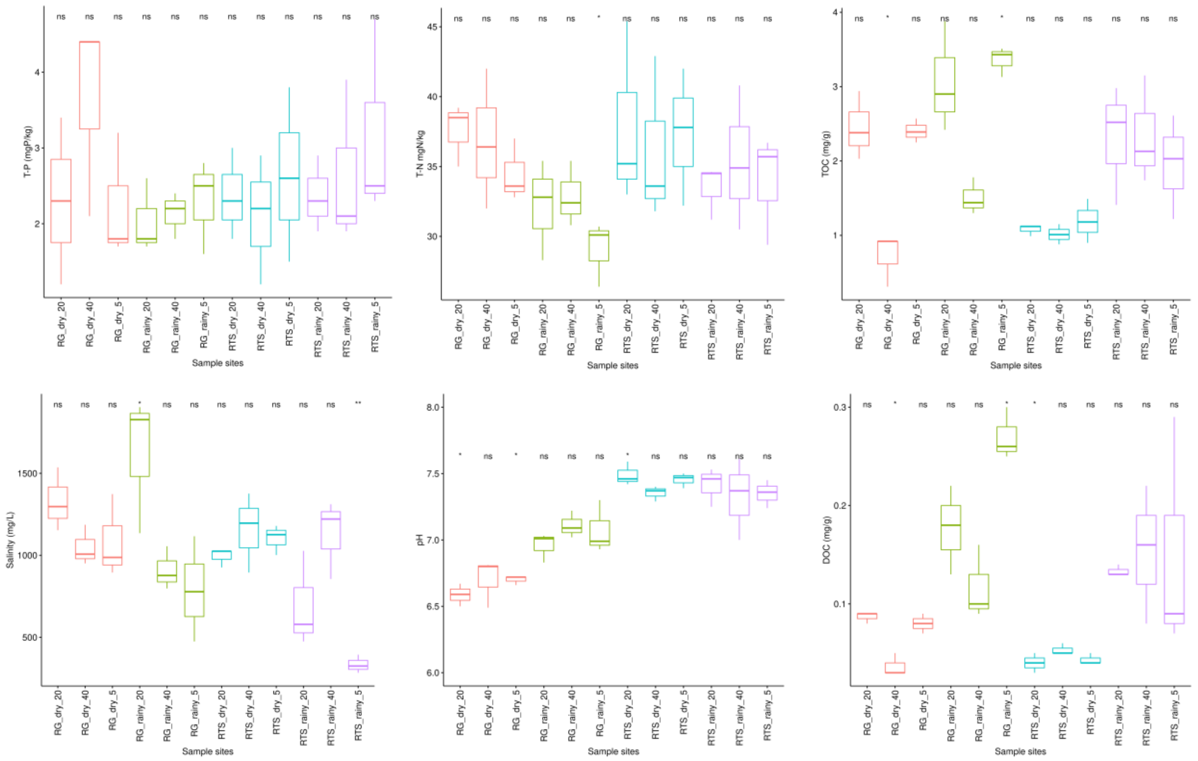

Fig. 3.

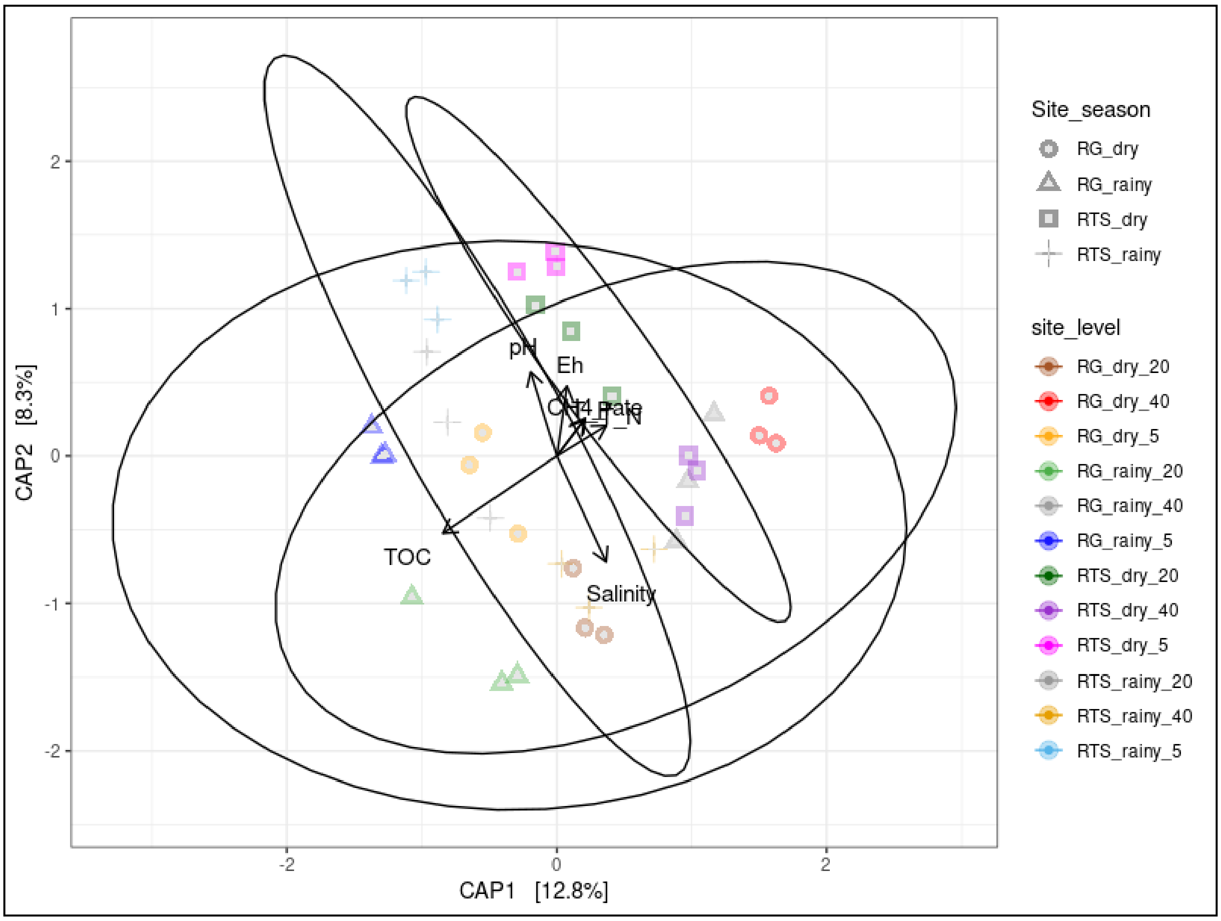

***VECTORS

	CCA1	CCA2	r2	Pr(>r)
pH	-0.69933	0.71480	0.2638	0.008 **
Salinity	0.72879	-0.68473	0.7159	0.001 ***
Eh	-0.30402	0.95267	0.2949	0.005 **
T_N	0.52507	0.85106	0.1836	0.029 *
T_P	0.14352	0.98965	0.0809	0.243
TOC	-0.57076	-0.82111	0.9520	0.001 ***
CH4_rate	0.43759	0.89917	0.1109	0.153

Signif. codes: 0 ‘***’ 0.001 ‘**’ 0.01 ‘*’ 0.05 ‘.’ 0.1 ‘ ’ 1

Permutation: free

Number of permutations: 999

Other information

Pairwise comparisons using Wilcoxon rank sum exact test

1) Shannon index *P*-values

Based on depths

	RG_dry_20	RG_dry_40	RG_dry_5	RG_rainy_20	RG_rainy_40	RG_rainy_5	RTS_dry_20	RTS_dry_40	RTS_dry_5	RTS_rainy_20	RTS_rainy_40
RG_dry_40	1	-	-	-	-	-	-	-	-	-	-
RG_dry_5	1	1	-	-	-	-	-	-	-	-	-
RG_rainy_20	1	1	1	-	-	-	-	-	-	-	-
RG_rainy_40	1	1	1	1	-	-	-	-	-	-	-
RG_rainy_5	1	1	1	1	1	-	-	-	-	-	-
RTS_dry_20	1	1	1	1	1	1	-	-	-	-	-
RTS_dry_40	1	1	1	1	1	1	1	-	-	-	-
RTS_dry_5	1	1	1	1	1	1	1	1	-	-	-
RTS_rainy_20	1	1	1	1	1	1	1	1	1	-	-
RTS_rainy_40	1	1	1	1	1	1	1	1	1	1	-
RTS_rainy_5	1	1	1	1	1	1	1	1	1	1	1

Based on seasons

	**RG_dry**	**RG_rainy**	**RTS_dry**
**RG_rainy**	**0.0311**	**-**	**-**
**RTS_dry**	**0.5162**	**0.0426**	**-**
**RTS_rainy**	**0.0047**	**0.5162**	**0.0047**

2) Chao index *P*-values

Based on depths

	RG_dry_20	RG_dry_40	RG_dry_5	RG_rainy_20	RG_rainy_40	RG_rainy_5	RTS_dry_20	RTS_dry_40	RTS_dry_5	RTS_rainy_20	RTS_rainy_40
RG_dry_40	1	-	-	-	-	-	-	-	-	-	-
RG_dry_5	1	1	-	-	-	-	-	-	-	-	-
RG_rainy_20	1	1	1	-	-	-	-	-	-	-	-
RG_rainy_40	1	1	1	1	-	-	-	-	-	-	-
RG_rainy_5	1	1	1	1	1	-	-	-	-	-	-
RTS_dry_20	1	1	1	1	1	1	-	-	-	-	-
RTS_dry_40	1	1	1	1	1	1	1	-	-	-	-
RTS_dry_5	1	1	1	1	1	1	1	1	-	-	-
RTS_rainy_20	1	1	1	1	1	1	1	1	1	-	-
RTS_rainy_40	1	1	1	1	1	1	1	1	1	1	-
RTS_rainy_5	1	1	1	1	1	1	1	1	1	1	1

Based on seasons

	RG_dry	RG_rainy	RTS_dry
RG_rainy	0.97886	-	-
RTS_dry	0.00099	0.02332	-
RTS_rainy	0.00099	0.02254	0.97886

3) InvSimpson index *P*-values

Based on depth

	RG_dry_20	RG_dry_40	RG_dry_5	RG_rainy_20	RG_rainy_40	RG_rainy_5	RTS_dry_20	RTS_dry_40	RTS_dry_5	RTS_rainy_20	RTS_rainy_40
RG_dry_40	1	-	-	-	-	-	-	-	-	-	-
RG_dry_5	1	1	-	-	-	-	-	-	-	-	-
RG_rainy_20	1	1	1	-	-	-	-	-	-	-	-
RG_rainy_40	1	1	1	1	-	-	-	-	-	-	-
RG_rainy_5	1	1	1	1	1	-	-	-	-	-	-
RTS_dry_20	1	1	1	1	1	1	-	-	-	-	-
RTS_dry_40	1	1	1	1	1	1	1	-	-	-	-
RTS_dry_5	1	1	1	1	1	1	1	1	-	-	-
RTS_rainy_20	1	1	1	1	1	1	1	1	1	-	-
RTS_rainy_40	1	1	1	1	1	1	1	1	1	1	-
RTS_rainy_5	1	1	1	1	1	1	1	1	1	1	1

Based on seasons

	RG_dry	RG_rainy	RTS_dry
RG_rainy	0.1509	-	-
RTS_dry	10.000	0.1259	-
RTS_rainy	0.0938	10.000	0.0047

Permutation test for adonis under reduced model

Terms added sequentially (first to last)

Permutation: free

Number of permutations: 999

adonis2(formula = phylose_36_clean_bray ~ Site_season, data = df_map_36_clean)

	Df	SumOfSqs	R2	F	Pr(>F)
Site_season	3	1.7005	0.22267	3.0556	0.001 ***
Residual	32	5.9362	0.77733		
Total	35	7.6366	1.00000		

Signif. Codes: 0 ‘***’ 0.001 ‘**’ 0.01 ‘*’ 0.05 ‘.’ 0.1 ‘ ’ 1

Permutation test for homogeneity of multivariate dispersions

Permutation: free

Number of permutations: 999

Response: Distances

	Df	Sum Sq	Mean Sq	F	N.Perm	Pr(>F)
Groups	3	0.01866	0.0062215	0.6065	999	0.613
Residuals	32	0.32827	0.0102585			

Adonis pairs

	pairs	Df	SumsOfSqs	F.Model	R2	p.value	p.adjusted	sig
1	RG_rainy vs RTS_rainy	1	0.3846175	1.604854	0.0911	0.083	0.498	
2	RG_rainy vs RG_dry	1	0.5135620	2.166378	0.1192	0.025	0.150	
3	RG_rainy vs RTS_dry	1	0.7441776	3.342680	0.17281	0.003	0.018	.
4	RTS_rainy vs RG_dry	1	0.7599776	3.349792	0.17311	0.002	0.012	.
5	RTS_rainy vs RTS_dry	1	0.5961246	2.806060	0.1492	0.004	0.024	.
6	RG_dry vs RTS_dry	1	0.4161783	1.983283	0.1102	0.041	0.246	

Signif. Codes: 0 '***' 0.001 '**' 0.01 '*' 0.05 '.' 0.1 ' ' 1

	pairs			Df	Sums OfSqs	F.Model	R2	p.value	p.adjusted	sig
1	RG_rainy_5	vs	RG_rainy_20	1	0.3468797	3.13523	0.4394014	0.1	1	
2	RG_rainy_5	vs	RG_rainy_40	1	0.8661578	6.532271	0.6202149	0.1	1	
3	RG_rainy_5	vs	RTS_rainy_5	1	0.262758	1.852622	0.3165456	0.1	1	
4	RG_rainy_5	vs	RTS_rainy_20	1	0.3184724	2.477774	0.3825039	0.1	1	
5	RG_rainy_5	vs	RTS_rainy_40	1	0.6894242	5.635307	0.5848602	0.1	1	
6	RG_rainy_5	vs	RG_dry_5	1	0.273646	2.665657	0.3999091	0.1	1	
7	RG_rainy_5	vs	RG_dry_20	1	0.5034438	4.917711	0.5514544	0.1	1	
8	RG_rainy_5	vs	RG_dry_40	1	0.9185022	7.53974	0.6533717	0.1	1	
9	RG_rainy_5	vs	RTS_dry_5	1	0.4511084	3.954096	0.4971144	0.1	1	
10	RG_rainy_5	vs	RTS_dry_20	1	0.4444511	3.691949	0.4799758	0.1	1	

11	RG_rainy_5	vs	RTS_dry_40	1	0.7141212	6.623202	0.6234657	0.1	1
12	RG_rainy_20	vs	RG_rainy_40	1	0.6106359	4.118766	0.5073143	0.1	1
13	RG_rainy_20	vs	RTS_rainy_5	1	0.4869054	3.091647	0.4359561	0.1	1
14	RG_rainy_20	vs	RTS_rainy_20	1	0.2844257	1.972549	0.3302692	0.1	1
15	RG_rainy_20	vs	RTS_rainy_40	1	0.3578392	2.59303	0.3932987	0.1	1
16	RG_rainy_20	vs	RG_dry_5	1	0.4286365	3.622798	0.4752583	0.1	1
17	RG_rainy_20	vs	RG_dry_20	1	0.2844551	2.409944	0.3759696	0.1	1
18	RG_rainy_20	vs	RG_dry_40	1	0.7620261	5.542743	0.5808333	0.1	1
19	RG_rainy_20	vs	RTS_dry_5	1	0.6831448	5.265222	0.568278	0.1	1
20	RG_rainy_20	vs	RTS_dry_20	1	0.5217309	3.835012	0.4894711	0.1	1
21	RG_rainy_20	vs	RTS_dry_40	1	0.5087447	4.12001	0.5073898	0.1	1
22	RG_rainy_40	vs	RTS_rainy_5	1	0.7893056	4.398519	0.5237255	0.1	1
23	RG_rainy_40	vs	RTS_rainy_20	1	0.6549272	3.941798	0.4963357	0.1	1
24	RG_rainy_40	vs	RTS_rainy_40	1	0.3857277	2.411434	0.3761146	0.1	1
25	RG_rainy_40	vs	RG_dry_5	1	0.8196652	5.843322	0.5936331	0.1	1
26	RG_rainy_40	vs	RG_dry_20	1	0.6092379	4.35197	0.5210711	0.1	1
27	RG_rainy_40	vs	RG_dry_40	1	0.3497436	2.193586	0.3541706	0.1	1
28	RG_rainy_40	vs	RTS_dry_5	1	0.801002	5.280031	0.5689669	0.1	1
29	RG_rainy_40	vs	RTS_dry_20	1	0.6584842	4.167581	0.5102589	0.1	1
30	RG_rainy_40	vs	RTS_dry_40	1	0.4194258	2.883864	0.418931	0.1	1

31	RTS_rainy_5	vs	RTS_rainy_20	1	0.2326183	1.326345	0.2490159	0.3	1
32	RTS_rainy_5	vs	RTS_rainy_40	1	0.6330384	3.741551	0.4833077	0.1	1
33	RTS_rainy_5	vs	RG_dry_5	1	0.4135623	2.766165	0.4088232	0.1	1
34	RTS_rainy_5	vs	RG_dry_20	1	0.5943342	3.982808	0.4989232	0.1	1
35	RTS_rainy_5	vs	RG_dry_40	1	0.8584021	5.089156	0.5599151	0.1	1
36	RTS_rainy_5	vs	RTS_dry_5	1	0.3281896	2.039234	0.3376644	0.1	1
37	RTS_rainy_5	vs	RTS_dry_20	1	0.3540431	2.117037	0.3460886	0.1	1
38	RTS_rainy_5	vs	RTS_dry_40	1	0.6650139	4.299498	0.5180431	0.1	1
39	RTS_rainy_20	vs	RTS_rainy_40	1	0.3866028	2.479928	0.3827092	0.1	1
40	RTS_rainy_20	vs	RG_dry_5	1	0.4245403	3.116836	0.4379525	0.1	1
41	RTS_rainy_20	vs	RG_dry_20	1	0.415838	3.059292	0.4333709	0.1	1
42	RTS_rainy_20	vs	RG_dry_40	1	0.7672703	4.938213	0.5524832	0.1	1
43	RTS_rainy_20	vs	RTS_dry_5	1	0.4788461	3.243358	0.44777	0.1	1
44	RTS_rainy_20	vs	RTS_dry_20	1	0.3112577	2.021988	0.3357675	0.1	1
45	RTS_rainy_20	vs	RTS_dry_40	1	0.4739122	3.352194	0.4559447	0.1	1
46	RTS_rainy_40	vs	RG_dry_5	1	0.6517622	5.012892	0.5561913	0.1	1
47	RTS_rainy_40	vs	RG_dry_20	1	0.4237726	3.266456	0.4495253	0.1	1
48	RTS_rainy_40	vs	RG_dry_40	1	0.567707	3.805452	0.4875377	0.1	1
49	RTS_rainy_4	vs	RTS_dry_5	1	0.7252995	5.127697	0.5617734	0.1	1

0

50	RTS_rainy_40	vs	RTS_dry_20	1	0.556741	3.768258	0.485084	0.1	1
51	RTS_rainy_40	vs	RTS_dry_40	1	0.2933371	2.169939	0.3516953	0.1	1
52	RG_dry_5	vs	RG_dry_20	1	0.2986831	2.71405	0.4042344	0.1	1
53	RG_dry_5	vs	RG_dry_40	1	0.7358674	5.682437	0.5868809	0.1	1
54	RG_dry_5	vs	RTS_dry_5	1	0.3270838	2.686224	0.401755	0.1	1
55	RG_dry_5	vs	RTS_dry_20	1	0.3111239	2.429499	0.3778675	0.1	1
56	RG_dry_5	vs	RTS_dry_40	1	0.5454199	4.722322	0.5414065	0.1	1
57	RG_dry_20	vs	RG_dry_40	1	0.5504462	4.259891	0.5157321	0.1	1
58	RG_dry_20	vs	RTS_dry_5	1	0.5354378	4.407588	0.5242393	0.1	1
59	RG_dry_20	vs	RTS_dry_20	1	0.3581008	2.802514	0.4119821	0.1	1
60	RG_dry_20	vs	RTS_dry_40	1	0.3044257	2.642224	0.397792	0.1	1
61	RG_dry_40	vs	RTS_dry_5	1	0.7003067	4.969225	0.5540306	0.1	1
62	RG_dry_40	vs	RTS_dry_20	1	0.5622518	3.818964	0.4884232	0.1	1
63	RG_dry_40	vs	RTS_dry_40	1	0.3353882	2.490563	0.3837207	0.1	1
64	RTS_dry_5	vs	RTS_dry_20	1	0.2409573	1.727402	0.3016031	0.1	1
65	RTS_dry_5	vs	RTS_dry_40	1	0.5570574	4.388751	0.523171	0.1	1
66	RTS_dry_20	vs	RTS_dry_40	1	0.3479922	2.612045	0.3950435	0.1	1

pairs	Df	SumsOfSqs	F.Model	R2	p.value	p.adjusted	sig

1	RG vs RTS	1	0.5976322	2.447625	0.06715457	0.008	0.008 *

	pairs	Df	SumsOfSqs	F.Model	R2	p.value	p.adjusted	sig
1	5 vs 20	1	0.6125904	3.164849	0.1257647	0.001	0.003	*
2	5 vs 40	1	1.8156671	9.003241	0.2903968	0.001	0.003	*
3	20 vs 40	1	1.0020432	4.866504	0.1811365	0.001	0.003	*
